# A simple set of validation steps identifies and removes false results in a sandwich enzyme-linked immunosorbent assay caused by anti-animal IgG antibodies in plasma from arthritis patients

**DOI:** 10.1186/2193-1801-2-263

**Published:** 2013-06-15

**Authors:** Tue W Kragstrup, Thomas Vorup-Jensen, Bent Deleuran, Malene Hvid

**Affiliations:** Department of Biomedicine, Aarhus University, Wilhelm Meyers Allé 4, Aarhus C, DK-8000 Denmark; Department of Clinical Medicine, Aarhus University, Brendstrupgårdsvej 100, Aarhus N, DK-8200 Denmark; Department of Rheumatology, Aarhus University Hospital, Nørrebrogade 44, Aarhus C, DK-8000 Denmark

**Keywords:** Arthritis, Enzyme-linked immunosorbent assay, Immunoassay, Multiplex, Interference, Rheumatoid factor, Anti-animal IgG antibodies, Heterophilic antibodies, ELAST amplification system

## Abstract

Rheumatoid arthritis (RA) and spondyloarthritis (SpA) are chronic diseases characterized by activation of the immune system and production of antibodies. Thus, rheumatoid factor, anti-animal IgG antibodies and heterophilic antibodies in plasma samples from arthritis patients can interfere with immunoassays such as sandwich enzyme-linked immunosorbent assay (ELISA) systems often used in arthritis research. However, standard methodologies on how to test for false results caused by these antibodies are lacking. The objective of this study was to design a simple set of steps to validate a sandwich ELISA before using it for measuring analytes in plasma from arthritis patients. An interleukin-24 (IL-24) sandwich ELISA system was prepared with a monoclonal mouse capture antibody and a polyclonal goat detection antibody and tested for interference by rheumatoid factor, anti-animal IgG antibodies and heterophilic antibodies. Plasma samples from 23 patients with RA and SpA were used. No differences were found between plasma samples measured in wells coated with anti-IL-24 specific antibody and in wells coated with isotype control antibody (false positive results), and recombinant human IL-24 was not recovered in spiked samples (false negative results). This interference was removed after preincubating the plasma samples from patients with arthritis with goat or bovine IgG, suggesting that anti-animal IgG antibodies found in the plasma of the arthritis patients caused the false results. Additional testing showed that the signal-to-noise ratio could be increased by titration of the capture and detection antibodies and by using the ELAST amplification system. Finally, the calculated concentration of IL-24 was increased in ethylenediaminetetraacetic acid (EDTA) plasma compared to heparin plasma and serum and decreased with repetitive freeze/thaw cycles of the samples illustrating how sample handling could additionally contribute to the variations reported by different laboratories in measurement of the same analyte. This study proposes a simple set of validation steps to evaluate and optimize a sandwich ELISA before using it for measuring analytes in plasma from arthritis patients. Anti-animal IgG antibodies are also present in healthy individuals, suggesting that validation of ELISA systems for measuring non-arthritis samples could also be improved by this simple set of validation steps.

## Background

Rheumatoid arthritis (RA) and spondyloarthritis (SpA) are chronic inflammatory diseases characterized by diffuse activation of leukocytes and production of antibodies (Dougados and Baeten 2011; McInnes and Schett 2011). These antibodies are autoantibodies, anti-animal protein antibodies and heterophilic antibodies (Bartels and Ribel-Madsen 2013). Autoantibodies are antibodies to immunological self-antigens, in the human body typically other proteins of an endogenous origin. Rheumatoid factor is an autoantibody against the fragment crystallizable region (Fc region) of human IgG and is a characteristic of RA but can also increase in healthy individuals during an infection (Ball and Lawrence 1961; Waaler 1940; Welch et al. 1983). Several other autoantibodies are found in both RA and SpA patients (Duskin and Eisenberg 2010; Wright et al. 2012). Anti-animal protein antibodies are antibodies to animal proteins, e.g. animal IgG (Degn et al. 2011; Husby et al. 1985). These antibodies are prevalent in plasma from patients with both RA and SpA and in plasma from healthy individuals. Human anti-animal IgG antibodies can arise from multiple sources including blood transfusion, vaccination or transfer of dietary antigens across the gut wall (Andersen et al. 2004; Hawkins et al. 1980; Jewell and Truelove 1972). One study reported such antibodies to be found in 95% of plasma samples collected from healthy individuals (Andersen et al. 2004). Heterophilic antibodies are weak and polyspecific antibodies. These antibodies are found in healthy individuals as natural antibodies inherently produced by the immune system and are increased in autoimmune disease (Levinson and Miller 2002).

Rheumatoid factor, anti-animal IgG antibodies and heterophilic antibodies can be a major problem in immunoassays such as sandwich enzyme-linked immunosorbent assay (ELISA) systems. In particular human rheumatoid factor is notorious for its ability to bind the Fc region of IgG from nearly any species (Hamilton et al. 1988). The sandwich ELISA is usually performed by first immobilizing a capture antibody in polystyrene wells, then adding the sample containing the analyte and finally developing a reaction with a detection antibody conjugated to an enzyme. Usually these antibodies are monoclonal mouse or rat antibodies or purified polyclonal antibodies from rabbits or goats. False positive results can be caused if rheumatoid factor, anti-animal IgG antibodies or heterophilic antibodies bridge the capture and detection antibodies to yield a signal even in the absence of analyte (Bartels et al. 2011; Ismail et al. 2002; Levinson and Miller 2002; Selby 1999). False negative results can be caused if these antibodies react with either the capture antibody or the detection antibody preventing reaction with the analyte (Kricka 1999). Anti-animal IgG antibodies are a problem, especially when monoclonal antibodies are used in the sandwich ELISA. Thus monoclonal antibodies made by hybridomas cultured in medium with fetal calf serum (FCS) could be contaminated with bovine IgG. This contamination occurs because commercially available FCS contains bovine IgG contaminating the hybridoma supernatant. Purification of the IgG fraction of the hybridoma supernatant on a protein A or G column is not species specific, and both monoclonal antibody and bovine IgG are collected (Goudswaard et al. 1978). The contaminating bovine IgG has been reported to comprise up to 95% of the immunoglobulin in the hybridoma supernatant (Harlow and Lane 1988). Human anti-bovine IgG antibodies in the sample to be measured can thus bridge contaminating bovine IgG in the capture and detection mouse antibody solutions. Also anti-bovine IgG antibodies often cross-react with especially goat IgG due to species similarities. This can cause problems in sandwich ELISA systems with a combination of monoclonal mouse and polyclonal goat antibodies. There are several other factors influencing the binding of capture and detection antibodies to the analyte, e.g. presence of soluble receptors (Engelberts et al. 1991).

Immunoassays are used both in the clinical setting and for research. The problem with interfering rheumatoid factor, anti-animal IgG antibodies and heterophilic antibodies is generally accepted and different recommendations have been made to reduce false positive results. Thus, interference can be diminished by preincubating the samples with animal serum, animal immunoglobulin or a commercially available blocking agent or by using protein precipitation with either polyethylene glycol or Protein A, G or L (Bartels and Ribel-Madsen 2013). Interference in clinical diagnostic immunoassays may result in misdiagnosis and mistreatment (Sturgeon and Viljoen 2011). Thus, diagnostic companies put a lot of effort into preventing and controlling it. In the research setting, recommendations on how to make a simple validation of a sandwich ELISA for measuring plasma samples are still lacking. Thus, both commercially available and in-house sandwich ELISA systems and multiplex systems are frequently used with the risk of getting false results (Churchman et al. 2012; de Jager et al. 2005; DeForge et al. 2010; Martins et al. 2004; Todd et al. 2011).

Interleukin-24 (IL-24) is a member of the IL-10 family of cytokines comprising IL-10, IL-19, IL-20, IL-22, IL-24, IL-26, IL-28, and IL-29 sharing amino acid identity and protein structure (Ouyang et al. 2011). Furthermore, IL-19, IL-20 and IL-24 share the same two receptor complexes of IL-20R1/IL-20R2 and IL-22R/IL-20R2. IL-24 is primarily believed to be a tumor suppressor but has also been described in RA and SpA (Dash et al. 2010; Kragstrup et al. 2008).

This study establishes a simple set of steps to validate and optimize a sandwich ELISA before using it for measuring plasma from arthritis patients using an anti-IL-24 sandwich ELISA system as an example consisting of a mouse capture antibody and a goat detection antibody.

## Results

### Preparing the ELISA system

**Figure 1:**
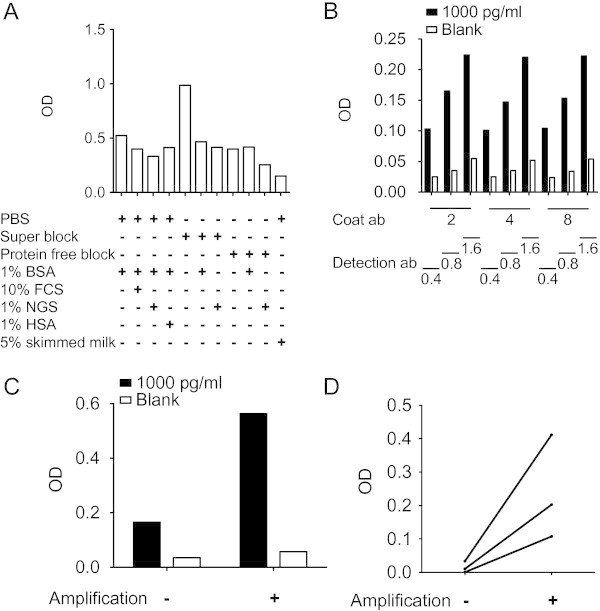
**Preparing the sandwich ELISA system.** (**A**) Testing different blockers of non-specific binding sites in polystyrene wells. OD signal from wells with blank assay diluent after blocking the wells with different blocking reagents. (**B**) Titration of capture and detection antibodies. OD signal from wells with blank assay diluent (noise) and wells with 1000 pg/ml rh IL-24 in assay diluent (signal) using different concentrations of capture antibody and detection antibody. Numbers represent concentrations in μg/ml. (**C**) OD signal from wells with blank assay diluent (noise) and wells with 1000 pg/ml rh IL-24 in assay diluent (signal) without or with amplification. (**D**) OD signal from wells with plasma samples from three different arthritis patients without or with amplification.

The sandwich ELISA used as an example in this study was a sandwich ELISA directed against the cytokine IL-24. The capture antibody was a protein A or G purified mouse anti-IL-24 monoclonal IgG2a antibody and the detection antibody was a goat anti-IL-24 polyclonal antibody. First, measuring the optical density (OD) signal from blank wells with diluent after blocking the non-specific binding sites in the polystyrene wells examined the best blocking reagent. Phosphate buffered saline (PBS) supplemented with 5% skimmed milk reduced the background noise most efficiently (Figure 1A).

Then, the signal-to-noise ratio was optimized. Both capture and detection antibodies were titrated at three different concentrations measuring blank assay diluent (noise) and assay diluent with 1000 pg/ml of recombinant human (rh) IL-24 (signal). The optimal concentrations were found to be 2 μg/ml for the capture antibody and 0.8 μg/ml for the detection antibody (Figure 1B). Furthermore, an amplification step with biotinyl-tyramide was tested. This increased the signal-to-noise ratio from 4.7 to 9.9 increasing the sensitivity of the assay (Figures 1C and 1D).

### Validating the ELISA system for measuring plasma from arthritis patients by testing for false positive results, false negative results and other matrix effects

**Figure 2:**
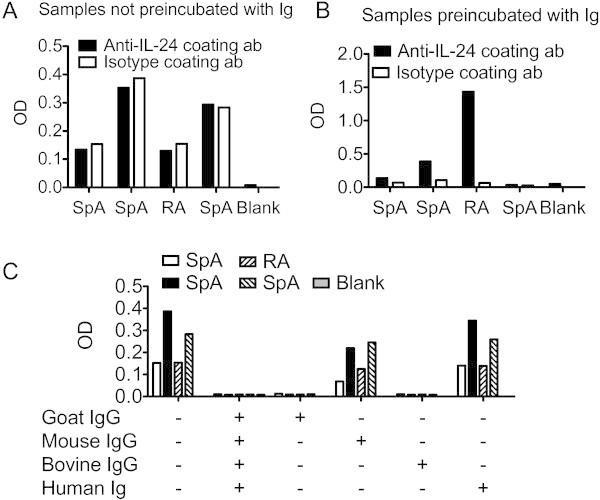
**Testing for false positive results.** (**A**) False positive measurements. OD signal from four patient samples in wells coated with anti-IL-24 specific antibody or with isotype control antibody. (**B**) Identification and removal of false positive measurements. OD signal from the same four patient samples in wells coated with anti-IL-24 specific antibody or with isotype control antibody after preincubating samples with a combination of mouse, goat, bovine and human immunoglobulins. (**C**) Identification of species specificity of the anti-animal IgG antibodies in the patient samples. OD signal from the same four patient samples in wells coated with isotype antibody after preincubating samples with different immunoglobulins as indicated.

Sandwich ELISA analysis of samples from RA and SpA patients are often complicated by the presence of rheumatoid factor, anti-animal IgG antibodies or heterophilic antibodies. First, an analysis to identify false positive results caused by these factors was conducted. Thus, to identify false positive results plasma samples from patients with arthritis were both measured in wells coated with anti-IL-24 specific antibody and in wells coated with isotype matched control antibody in an identical concentration. OD readings were similar in wells coated with isotype antibody compared with wells coated with antigen specific antibody indicating false positive results (Figure 2A). To reduce the amount of unspecific binding, the plasma samples from patients with arthritis were preincubated with a combination of mouse, goat, bovine and human immunoglobulins. Preincubation with immunoglobulins lowered the OD readings in wells coated with isotype antibody without eliminating the true OD readings in wells coated with antigen specific antibody (Figure 2B). To further determine the origin of the interfering antibodies, samples were incubated with either mouse IgG, goat IgG, bovine IgG or human immunoglobulin. There was a dramatic decrease in unspecific binding after adding bovine IgG or goat IgG with much less effect of adding mouse IgG or human immunoglobulin (Figure 2C) indicating that the antibodies interfering with the assay were anti-animal IgG antibodies with reactivity to goat and bovine IgG. Thus, false positive results were identified and reduced.

**Figure 3:**
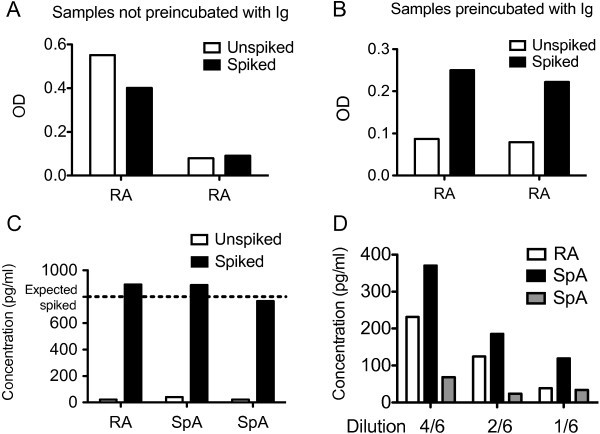
**Testing for false negative results and other matrix effects.** (**A**) False negative measurements. OD signal from unspiked and spiked plasma samples from two arthritis patients in wells coated with anti-IL-24 specific antibody. (**B**) Identification and removal of false negative measurements. OD signal from unspiked and spiked plasma samples from the same two arthritis patients in wells coated with anti-IL-24 specific antibody after preincubating samples with a combination of mouse, goat, bovine and human immunoglobulins. (**C**) Spike recovery assessment in three other arthritis patients. Calculated IL-24 concentrations from unspiked patient samples and patient samples spiked with 800 pg/ml rh IL-24 in wells coated with anti-IL-24 specific antibody after preincubating samples with immunoglobulins. (**D**) Linearity-of-dilution assessment in three other arthritis patients. Calculated IL-24 concentrations in samples preincubated with immunoglobulin and diluted 4/6, 2/6 and 1/6 in wells coated with anti-IL-24 specific antibody.

Then, possible inhibitors of the ELISA system producing false negative results were identified. Thus, the samples were spiked with rh IL-24 before and after preincubation with mouse, goat, bovine and human immunoglobulins. Spiked samples with recombinant cytokine only increased the OD readings in samples preincubated with immunoglobulin indicating false negative results (Figures 3A and 3B). Another three patient samples were spiked to an expected concentration of rh IL-24 of 800 pg/ml and recovery was calculated to be 108%, 106% and 93% (Figure 3C).

Finally, the sandwich ELISA was further tested for other matrix effects by diluting plasma samples from arthritis patients. The samples could be diluted 2-fold showing almost linearity until reaching a concentration of IL-24 of approximately 25 pg/ml (Figure 3D).

### Testing the ELISA system for variability, stability of analyte, effect of sample anticoagulant, effect of assay buffer content on the standard curve and cross-reactivity

**Figure 4:**
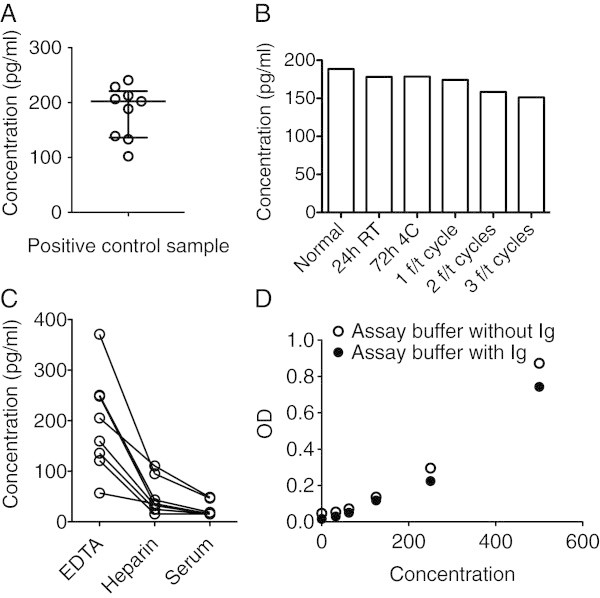
**Additional testing of the ELISA system.** (**A**) Testing the ELISA system for variability. Calculated IL-24 concentrations in the positive control sample analyzed in nine different experiments. (**B**) Testing the ELISA system for stability of the analyte. Calculated IL-24 concentrations in the positive control sample after storage under different conditions or after a variable number of freeze/thaw cycles (f/t). (**C**) Testing the ELISA system for effect of the sample anticoagulant used. Calculated IL-24 concentrations in eight paired plasma samples from patients with arthritis stabilized with either EDTA or heparin or serum samples. (**D**) Testing the ELISA system for effect on the standard curve of adding immunoglobulins to the assay diluent. OD signal from samples of rh IL-24 diluted in assay buffer without immunoglobulin and from samples of rh IL-24 diluted in assay buffer with immunoglobulin.

First, inter-assay variability was tested. A positive control sample was run in duplicates in different plates. The positive control sample was prepared by pooling ethylenediaminetetraacetic acid (EDTA) plasma from four healthy individuals identified to have non-detectable concentrations of IL-24 and adding rh IL-24 for a final concentration of 200 pg/ml. The inter-assay coefficient of variability was calculated to be 25% (Figure 4A).

Then the stability of the cytokine was examined. Different vials of the positive control plasma sample were stored under different conditions or exposed to freeze/thaw cycles. Repetitive freeze/thaw cycles resulted in small decreases in measured concentrations of IL-24 (Figure 4B).

Then, the effect of the anticoagulant used for the sample preparation on the calculated concentration of IL-24 was tested. Paired plasma samples stabilized with both EDTA and heparin and serum samples were analyzed. The concentration of measured IL-24 was best detected in plasma stabilized with EDTA (Figure 4C).

Adding high concentrations of immunoglobulin to the assay diluent can potentially alter the signals in the standard curve. Thus, standard curves were prepared using assay diluent with and without immunoglobulin. The standard curve showed a small decrease in OD signal after adding immunoglobulin to the assay diluent (Figure 4D). IL-24 shares amino acid sequence, protein structure and receptor subunits with IL-19 and IL-20. Therefore cross-reactivity to these two cytokines was tested. No cross-reactivity was detected (data not shown).

## Discussion

Sandwich ELISA systems are frequently used in arthritis research. Rheumatoid factor, anti-animal IgG antibodies and heterophilic antibodies are commonly found in human plasma and increased in arthritis patients and can cause interference in sandwich ELISA systems. Thus, validation of any sandwich ELISA planed for arthritis research is important. This study demonstrates false positive results in plasma from arthritis patients caused by anti-animal IgG antibodies and the interference was identified and removed by a simple set of validation steps.

An anti-IL-24 sandwich ELISA system was prepared with a mouse monoclonal capture antibody and a goat polyclonal detection antibody after titration of the antibodies. It seems evident that an in-house sandwich ELISA constructed by the researcher should be validated thoroughly but the following set of validation steps could easily be used in any commercially available sandwich ELISA. Thus, commercially available kits are not always constructed with the appropriate blockers in the assay diluent and often not optimized for measuring arthritis samples. Also, this type of testing should be considered using other immunoassays such as multiplex systems (Churchman et al. 2012).

The signal-to-noise ratio was increased by an amplification step. The ELAST amplification system is based on the reporter deposition technology (Bobrow et al. 1989). This technique could become an important tool to increase the sensitivity in ELISA systems in research because cytokines and chemokines are often found in low concentrations in the blood. The most efficient plate blocker was skimmed milk. Skimmed milk is a well-known blocker of plastic surfaces and is always a good possible candidate for further empirical testing in the particular sandwich ELISA system to be prepared (Vogt et al. 1987).

The results of the ELISA system were validated by a simple set of steps and anti-animal IgG antibodies were found to cause both false positive and false negative results. The false positive results were most probably caused by anti-bovine IgG antibodies found in the plasma of the arthritis patients cross binding contaminating bovine IgG in the monoclonal capture antibody solution and the polyclonal goat detection antibody. Thus, the unspecific binding to the capture isotype antibody was removed after preincubating the samples with either goat IgG or bovine IgG. Rheumatoid factor did not seem to be a problem in this sandwich ELISA, because preincubating the samples with human immunoglobulin did not decrease the unspecific binding. Though, it cannot be excluded that these patients were all rheumatoid factor negative. Preincubating the samples with mouse IgG also reduced the false positive results to a minor extent. This suggests that heterophilic antibodies with weak polyspecificity could also account for some of the interference. The false negative results could be a result of the anti-animal IgG antibody reacting with either the capture antibody or the detection antibody and preventing reaction with the analyte (Kricka 1999). Thus, the spiked rhIL-24 was only recovered after preincubating the samples with immunoglobulin. Also, the OD increased in some of the measured samples after preincubating the samples with immunoglobulin. After preincubating the samples with immunoglobulin dilution of three different plasma samples showed assay linearity indicating that the signal actually comes from detection of the analyte in the sandwich ELISA. Though, even after thorough optimization residual interference cannot be completely excluded. A final step would be to measure all samples both in wells coated with antigen specific antibody and in wells coated with isotype control antibody and calculate the difference (Gjelstrup et al. 2010).

The validation of any sandwich ELISA used in arthritis research is important and could be done in a simple setup as described in this study and summarized in Table 1. The tests could be done with 3–6 patients using only approximately one microplate and only little patient material. Reporting the results from such a validation would only require limited space in the materials and methods section. This study demonstrates false positive results in plasma from arthritis patients caused by anti-animal IgG antibodies and not rheumatoid factor. This suggests that the proposed validation setup should also be considered when establishing immunoassays for measuring non-arthritis samples.

**Table 1:** **A simple set of steps to validate a sandwich ELISA for research use**

Potential problem	Validation step
*The essential steps*	
**False positive measurements**	Measure samples in wells coated with antigen specific antibody and in wells coated with an isotype antibody. Use samples before and after preincubation with immunoglobulins or another blocking agent.
**False negative measurements**	Spike samples with a known concentration of analyte and calculate the recovery. Use spiked samples before and after preincubation with immunoglobulins or another blocking agent.
**Other matrix effects**	Measure serial dilutions of samples to test for linearity. Use samples before and after preincubation with immunoglobulins or another blocking agent.
*The extra steps*	
**Low signal-to-noise ratio**	Test different solutions for blocking the non-specific binding sites in the polystyrene wells. Titrate the antibodies. Test an amplification step.
**High variability or dissimilarities between laboratories**	Use a positive control sample in every plate. Test the effect of repetitive freeze/thaw cycles. Test the effect of different sample anticoagulants. Test different buffers for making the standard curve.

After the simple validation, the ELISA system was tested further for variability, stability of the analyte, effect of anticoagulant used in the sample preparation, effect on the standard curve of adding immunoglobulins to the assay diluent and cross-reactivity. The inter-assay coefficient of variability was calculated to be 25%. This is a somewhat high coefficient of variation, which must be considered when interpreting results from the ELISA system. EDTA plasma was superior to heparin plasma and serum when meassuring the concentration of IL-24 in arthritis blood samples. The reason for the limited detection in heparin plasma and serum is not known. However, the effect of sample anticoagulant on analyte detection has been shown for other cytokines previously (de Jager et al. 2009), and it illustrates the importance of choosing the appropriate anti-coagulant.

Repetitive freeze/thaw cycles seemed to decrease the concentration of recombinant human IL-24 in the positive control sample. In this system, it is thus important to consider the use of samples that have not been frozen and thawed repeatedly. In a sandwich ELISA the concentration of analyte in the measured samples are typically calculated from a standard curve. Thus, it is important that the samples and the standard curve are measured in a diluent with a similar matrix. In this sandwich ELISA system the addition of immunoglobulin to the assay diluent changed the standard curve slightly. These findings all illustrate ways in which different laboratories might find different concentrations of the same analyte.

The test for cross-reactivity was conducted because IL-24 shares amino acid sequence, protein structure and receptor subunits with IL-19 and IL-20. This could be relevant in other studies with cytokines or other analytes with overlapping structures or functions. All these extra tests should also be considered when optimizing a sandwich ELISA system for arthritis research.

## Conclusions

In conclusion, this study establishes an IL-24 sandwich ELISA system and validates it for measuring plasma from arthritis patients with a simple set of validation steps. Any sandwich ELISA to be used in arthritis research and non-arthritis research could be validated using similar methodology.

## Methods

### Samples

Blood samples from 23 patients with chronic RA or SpA with no registration apart from their diagnosis were used. From 8 of the patients paired plasma samples stabilized with either EDTA or heparin and serum samples were analyzed. From the remaining of the patients either plasma samples stabilized with EDTA or heparin were used. To test for false positive results samples from 4 different patients were used and to test for false negative results samples from 2 different patients were used. All RA patients were diagnosed in accordance with the American College of Rheumatology (ACR) 1987 classification criteria (Arnett et al. 1988). All SpA patients met the European Spondyloarthropathy Study Group (ESSG) criteria (Dougados et al. 1991). EDTA plasma samples were also collected from 4 healthy individuals to make a positive control sample. These plasma samples were all identified to have non-detectable concentrations of IL-24. They were pooled and rh IL-24 was added for a final concentration of 200 pg/ml. All samples were obtained after informed written consent according to the Declaration of Helsinki and approved by the Local Ethics Committee (project number 20050046).

### Antibodies and reagents

A protein A or G purified anti-IL-24 monoclonal mouse IgG2a antibody (clone number 283161, catalog number MAB19652, RnD Systems, Minneapolis, USA) was used as capture antibody and a biotin conjugated anti-IL-24 polyclonal goat antibody (catalog number BAF1965, RnD Systems) was used as detection antibody. Horseradish peroxidase (HRP) conjugated streptavidin (catalog number DY998, RnD Systems) was used for visualization of bound antibody and rh IL-24 (from catalog number DY1965, RnD systems) was used for creation of a standard curve for quantification of concentrations. Rh IL-19 (from catalog number DY1035, RnD Systems) and rh IL-20 (from catalog number DY1102, RnD Systems) were used to test for cross-reactivity to these cytokines. Unspecific binding was assessed using a mouse IgG2a isotype capture antibody (catalog number MAB003, RnD systems). Mouse, goat and bovine IgG for blocking potential anti-animal IgG antibodies in the samples to be measured were purchased from Jackson ImmunoResearch (catalog numbers 015-000-003, 005-000-003 and 001-000-003, West Grove, USA) and human immunoglobulin for blocking potential anti-human immunoglobulin antibodies in the samples to be measured were purchased from Behring (Beriglobulin, King of Prussia, USA). Buffers to block non-specific binding sites in polystyrene wells were prepared with PBS pH 7.4, bovine serum albumin (BSA) (catalog number 12659, Calbiochem, San Diego, USA), normal goat serum (NGS) (catalog number 31873, Thermo Scientific, Waltham, USA), FCS (catalog number 10270, Gibco, Life Technologies, Carlsbad, USA), HSA (Human-Albumin 20%, 05002–00002, Behring), Superblock PBS Blocking Buffer (product number 37515, Thermo Scientific) and Protein Free PBS Blocking Buffer (catalog number 37572, Thermo Scientific). Wash buffer was prepared with PBS pH 7.4 with 0.05% Tween-20. For amplification of the signal the ELAST ELISA Amplification System was used (catalog number NEP116001EA, PerkinElmer, Waltham, USA).

### Assay diluent

The assay diluent was prepared as Protein-free PBS Blocking Buffer with different combinations of 10 μg/ml mouse IgG, 10 μg/ml goat IgG, 10 μg/ml bovine IgG and 10 μg/ml human immunoglobulin (heat-aggregated at 63°C for 30 min). Samples, positive control and standards were preincubated in assay diluent for 30 min at room temperature (RT) before addition to the plate. Samples and positive controls were diluted 2/3 in assay diluent. Samples, positive controls and standards prepared in assay diluent were added in a volume of 100 μl and plates were incubated overnight at 4°C.

### Blocking non-specific binding sites in polystyrene wells

The best blocking agent was determined in wells with blank assay diluent by blocking the plate with 300 μl of blocking buffer and incubating at RT for 2 hours. The following blocking buffers were tested. PBS with 1% BSA, PBS with 1% BSA and 10% FCS, PBS with 1% BSA and 1% NGS, PBS with 1% BSA and 1% HSA, Superblock PBS Blocking Buffer, Superblock PBS Blocking Buffer with 1% BSA, Superblock PBS Blocking Buffer with 1% NGS, Protein-free PBS Blocking Buffer, Protein-free PBS Blocking Buffer with 1% BSA, Protein-free PBS Blocking Buffer with 1% NGS or PBS with 5% skimmed milk. The optimal blocking buffer was found to be PBS with 5% skimmed milk and this blocking buffer was used in the subsequent experiments.

### Wash

Preceding all steps each well was washed three times with PBS with 0.05% Tween-20.

### Capture and detection antibodies and streptavidin-HRP

For capture of analyte maxisorp microplates (Nunc, Roskilde, Denmark) were coated with 100 μl per well of capture antibody or isotype antibody and incubated overnight at RT. The capture antibody concentration was titrated using 2 μg/ml, 4 μg/ml and 8 μg/ml in PBS. The optimal concentration was found to be 2 μg/ml and for the subsequent experiments capture antibody or isotype antibody were used in this concentration. For detection of analyte 100 μl of biotinylated detection antibody was added to each well and plates were incubated 1 hour at RT. The detection antibody concentration was titrated using 0.4 μg/ml, 0.8 μg/ml and 1.6 μg/ml in Protein-free PBS Blocking Buffer. The optimal concentration was found to be 0.8 μg/ml and for the subsequent experiments detection antibody was used in this concentration. For enzyme delivery 100 μl of streptavidin-HRP diluted 1/200 was added to each well for 15 min at RT.

### Amplification and optical density measurement

For the amplification step plates were first incubated with 100 μl of the biotinyl-tyramide solution per well for 15 min at RT and then for 30 min at RT with 100 μl streptavidin-HRP diluted 1/1000. The amplification step was used for all subsequent experiments. The plates were then incubated with 100 μl of tetramethylbenzidine (TMB) one (Kementec diagnostics, Taastrup, Denmark) per well at RT. Color development was stopped with 50 μl of 1 M H_2_SO_4_ per well. The optical density of each well was measured using a microplate reader set to 450 nm and wavelength correction set to 630 nm.

### Spike recovery and cross-reactivity

Samples were tested for spike recovery by adding rh IL-24 to plasma samples from patients with arthritis. The prepared IL-24 sandwich ELISA system was tested for cross-reactivity to IL-19 and IL-20 by adding rh IL-19 and rh IL-20 to assay diluent.

### Calculations

Results are reported as either OD signals or concentrations calculated from a standard curve using rh IL-24. The coefficient of variation was calculated as the standard deviation of the positive control (SD) divided by the mean of the positive control (mean) multiplied by 100% with the same positive control sample measured in nine different experiments.

The recovery of spiked rh IL-24 was calculated as the observed concentration of spiked sample (observed spiked) minus the observed concentration of the unspiked sample (observed unspiked) divided by the expected concentration of the spiked sample (expected spiked) multiplied by 100%.

Graphs were made with GraphPad Prism version 5 (GraphPad Software, San Diego, CA).

## Abbreviations

RA: Rheumatoid arthritis
SpA: Spondyloarthritis
IL: Interleukin
IgG: Immunoglobulin G
FCS: Fetal calf serum
ELISA: Enzyme-linked immunosorbent assay
ELAST: ELISA amplification system
EDTA: Ethylenediaminetetraacetic acid
PBS: Phosphate buffered saline
Rh: Recombinant human
Fc region: Fragment crystallizable region
OD: Optical density
BSA: Bovine serum albumin
HRP: Horseradish peroxidase
RT: Room temperature
TMB: Tetramethylbenzidine.
